# Higher HIV RNA Viral Load in Recent Patients with Symptomatic Acute HIV Infection in Lyon University Hospitals

**DOI:** 10.1371/journal.pone.0146978

**Published:** 2016-01-22

**Authors:** Isabelle Girerd-Genessay, Dominique Baratin, Tristan Ferry, Christian Chidiac, Vincent Ronin, Philippe Vanhems

**Affiliations:** 1 Infection Control and Epidemiology Unit, Hôpital Edouard Herriot, Hospices Civils de Lyon, Lyon, France; 2 Department of Infectious Diseases, Hospices Civils de Lyon, Lyon, France; 3 Infection Control and Epidemiology Unit, Hôpital Croix-Rousse, Hospices Civils de Lyon, Lyon, France; 4 Emerging Pathogens Laboratory, Fondation Mérieux, Centre International de Recherche en Infectiologie, INSERM U1111, CNRS UMR5308, ENS de Lyon, UCBL1, 21, Avenue Tony Garnier, Lyon, 69007, France; University of Pittsburgh Center for Vaccine Research, UNITED STATES

## Abstract

**Introduction:**

Increased human immunodeficiency virus (HIV) virulence at infection has been suggested by a meta-analysis based on viral load and CD4 T lymphocytes (CD4) count during acute infection. This result was obtained after secondary analyses of large databases, facilitating the detection of differences. Similar finding in cohorts of more modest sample size would indicate that the effect could be more substantial.

**Methods:**

Change from initial CD4 count and HIV viral load after acute HIV infection by calendar year was explored in patients treated at Lyon University hospitals. All patients admitted to our hospitals with acute HIV infection between 1996 and 2013 were included in our study. Initial CD4 count and viral load before the start of anti-retroviral treatment were analyzed. Trends over time were assessed in linear models.

**Results:**

Initial CD4 count remained similar over time. However, in 2006–2013, initial viral load rose significantly (+1.12 log_10_/ml/year, p = 0.01).

**Conclusion:**

Our data, obtained from a single hospital cohort, confirmed findings from a large meta-analysis, showed increased initial viremia at acute HIV infection since 2006 and suggesting potentially higher HIV virulence in recent years.

## Introduction

In 2010 in France, the number of individuals infected by HIV rose to around 150,000 [1] but decreased to 9,000 and 7,000 new cases diagnosed between 2003 and 2008, respectively [2]. HIV incidence declined from 9,000 new cases in 2003 to 6,300 in 2012 [3]. In 2012, 12% of new HIV infection cases were diagnosed as primary infections (PI), representing a significant increase since 2011. Several studies investigated potential changes in HIV virulence at PI across time on the basis of HIV RNA and CD4 measurements in blood. Virulence was approximated with low-level initial CD4 count and higher initial viral load [4–6].

Despite contradictory results, these studies were reviewed in depth in a recent meta-analysis [7] which pointed to a trend towards increased virulence, linked with early CD4 counts in 21,052 patients, and HIV viremia in 10,785 subjects. Indeed, the authors found a CD4 count loss of 4.93 cells/mm^3^/years (p<10^−4^) and viral load increase of 0.018 log_10_ copies/ml/years (p = 0.03), between 1984 and 2010. The large sample sizes facilitated the detection of statistical significance, and even modest outcomes. However, since the effect (i.e., higher HIV virulence at PI) remained high, it could be detected in cohorts with lower sample size. Our objective was to track a potential trend towards lower CD4 counts and higher HIV viral load at PI by year in our hospitals.

## Methods

All patients between 1996 and 2013 with documented acute HIV infections were found in the Lyon section of the French Hospital database based on previously-reported criteria [8]. This database has been approved by the national commission on computerized data and freedom (CNIL [*Commission Nationale de l'Informatique et des Libertés*] and the ethic advisory board) [9–10]. The patients were included in the database with anonymous number after giving written informed consent [11]. As observational study, no particular approval was necessary. Acute HIV infection was diagnosed as patients with a seroconversion biological proof (incomplete Western-Blot or quantitative PCR) or a positive serology in 2 years following a documented negative serology. Initial CD4 count and initial viral load (HIV RNA) were defined as the first measurements taken after the diagnosis of HIV infection. Only data collected before treatment started and within 3 months after HIV seroconversion were analyzed. Multiple linear regression assessed the evolution of initial CD4 count and initial viral load, according to a step-down regression with a 5% degree of significance.

## Results

**Fig 1:**
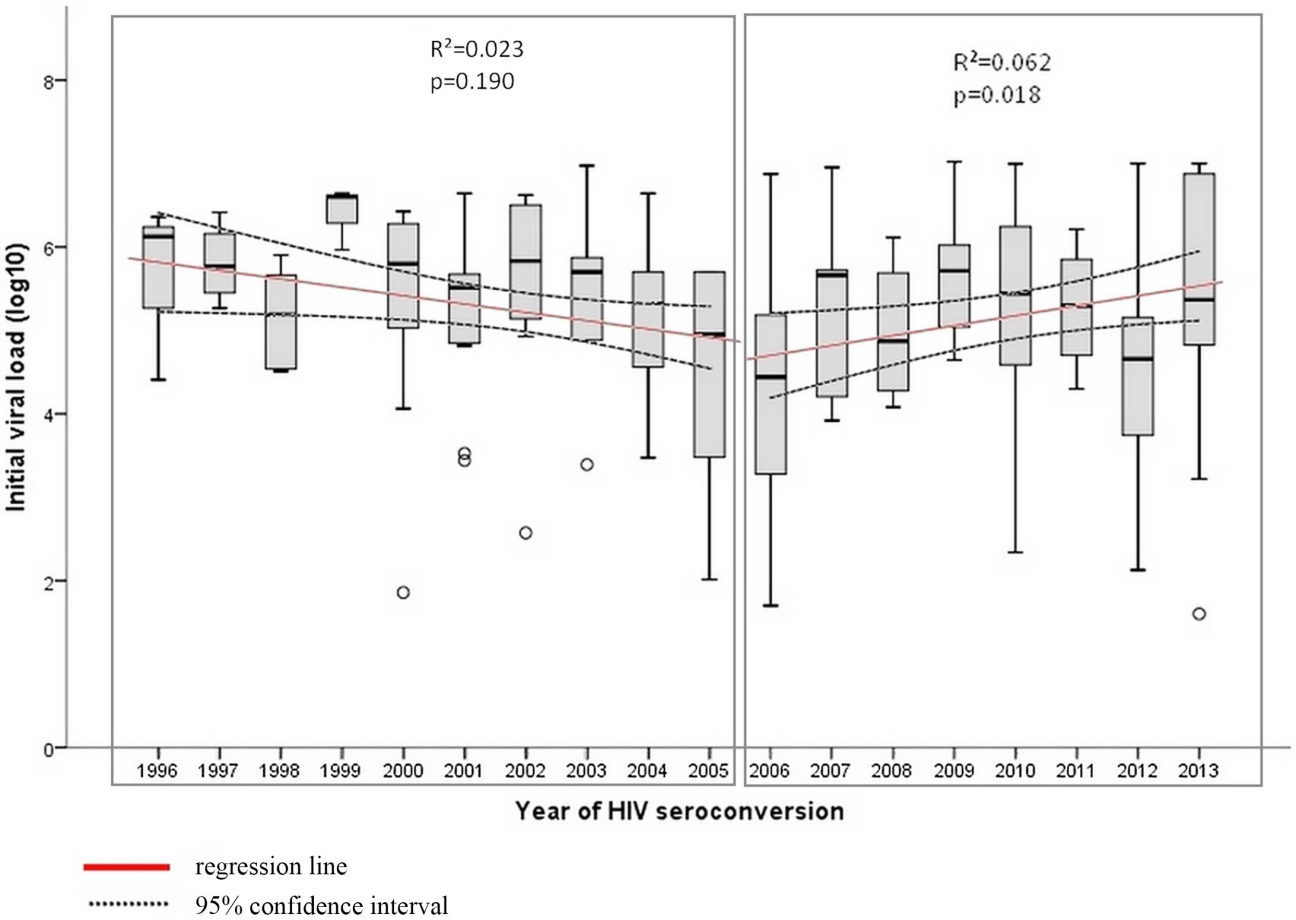
Initial viral load during acute HIV infection by year, Lyon university Hospitals, 1996 to 2013.

A total of 291 patients with documented PI between 1996 and 2013 were included in the cohort. All were HIV-1. Initial CD4 count before treatment started was available for 180 patients. Average CD4 count was 450/mm^3^ blood (±183.7), median was 427 (Interquartile Range IQR 259.3) and range was 95–959/mm^3^. Initial viral load before treatment started was known for 179 patients. Average HIV viremia was 5.23 log_10_ copies/ml plasma (SD 1.24) and median was 5.35 log_10_ copies/ml (IQR 1.5). Initial viral load ranged from 1.6 to 7.02 log_10_ copies/ml (S1 Fig). No trend in initial CD4 count was seen between 1996 and 2013, by linear regression, after adjustment for the time period between PI and measurement (-0.005 cells/mm^3^/year, p = 0.38) (S2 and S3 Figs). Initial viral load by time period seemed to comprise two stages: a decrease in the early 2000s, followed by an increase starting in 2006 (Fig 1, boxplot explanation is available on S4 Fig). From 1996 to 2005, no significant trend was detected after adjustment for the period between PI and measurement as well as for initial CD4 count (-0.66 log_10_/ml/year, p = 0.29). From 2006 to 2013, a significant increase was evident after adjustment for the period between PI and measurement and for initial CD4 count (+1.12 log_10_/ml/year, p = 0.01). These results indicate that, after 2006, HIV virulence might have changed or, at the very least, higher HIV RNA levels were detected after PI since 2006.

## Discussion

Divergence was observed in the evolution of prognostic markers, despite their correlation in our study. Indeed, no trend in initial CD4 count was emphasized by our investigation, contrary to results of the meta-analysis [7]. A loss of 4.9 cells/mm^3^/year since the outbreak started was assessed by the meta-analysis. However, our patient sample was relatively small and may have lacked power to detect statistical differences. On the other hand, increased initial viral loads were observed between 2006 and 2013. This result was highly significant (p = 0.01). Such an effect, detected in a cohort of modest sample size, would suggest a change in early pathogenetic events during PI, as it has been postulated elsewher [7]. It is compatible with the meta-analysis [7], which pointed to a trend towards increased virulence, linked with early HIV viremia in 10,785 subjects, showing an increase of 0.013 log_10_/ml/year, and supplements its results. Studies can detect all the more a small effect as their sample size is big, as this meta-analysis. Then, observing a similar effect in a much smaller sample as our study is a strong argument for the importance of the found effect. Indeed, we analyzed more recent data than those reviewed in the meta-analysis. They support the hypothesis of a constant effect over time. However, the trend encountered in our study was considerably higher than that assessed in the meta-analysis, so interpretation must proceed with caution.

An other study, analysing the data of about 16.000 patients, showed a trend to decreasing CD4 count at seroconversion and increasing initial viral load between the 1980s and 2002 [12]. No more trend was observed after 2002. Results observed from larger cohorts might mask some effects associated with local or country characteristics.

This observation leans towards growing virulence, as described previously [7]. Viral load seems to be a better prognosis factor than CD4 count [13]. However other interpretations are possible. We did not know the status of the contaminating person for each patient, and especially if viral inoculum size increased with time [14–16]. We didn't know exactly the seroconversion date, or viral load is highest in the first month after contamination. So, it would be an overall increase in average viral loads if patients are diagnosed earlier. However, we have no arguments in favour of an earlier diagnosis of the patients and there was no difference of average time between seroconversion and measure over time.

Disease stage at the time of virus transmission could be a confounding factor. Indeed, viral loads were particularly high during PI, then decreased before increasing again during later stages of the disease [17]. The viral load transmitted will be higher during these stages [18, 19]. Patients who do not know their current status and present greater viral loads than in the past, expose their sexual partners to higher inocula [20] which may result in PI with augmented viral loads. Similarly, evaluation of sexual behaviors in the last decade should be explored to assess potential influence on exposure to higher viral inocula.

Finally, time between seroconversion and diagnosis could be a confounding factor, since viral load is highest during the first weeks of the disease. However, systematic consideration of time between infection and viral load measurement in our multivariate analysis allows us to take this significant fluctuation into account during the first weeks of the disease and thus to control for the confounding effect. Increased initial viral load could also suggest more replication but without more virulence. To better assess HIV virulence, it is important to know patient outcomes, especially their evolution to AIDS or death.

In conclusion, our highly significant study, albeit with modest sample size, strengthens the hypothesis of increasing HIV virulence based on initial viral load measurement and in accordance with “big” cohort data [7].

## Supporting Information

S1 Fig Initial viral load during acute HIV infection by year, Lyon university Hospitals, 1996 to 2013. (TIFF)

S2 Fig Initial CD4 count during acute HIV infection by year, Lyon university Hospitals, 1996 to 2013. (TIFF)

S3 Fig Initial CD4 count during acute HIV infection by year, Lyon university Hospitals, 1996 to 2013. (TIFF)

S4 Fig Boxplot explanation. (TIFF)
