# Supplementary figures and images for: Higher HIV RNA Viral Load in Recent Patients with Symptomatic Acute HIV Infection in Lyon University Hospitals
Source: PLoS One. 2016 Jan 22;11(1):e0146978. doi: 10.1371/journal.pone.0146978 (PMC4723228; doi:10.1371/journal.pone.0146978)

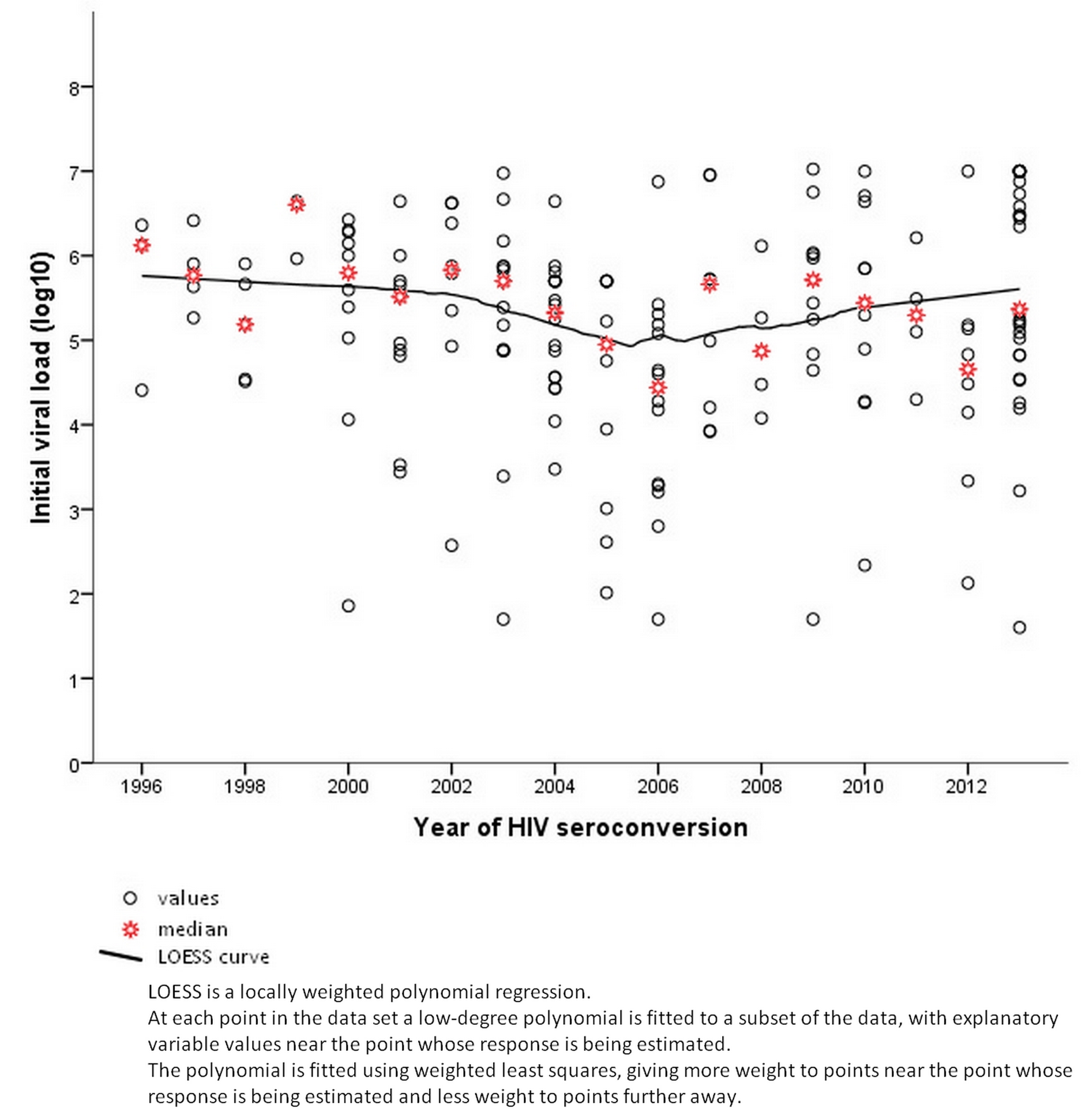

Supplement: S1 Fig — (TIFF) [file pone.0146978.s001.tiff]

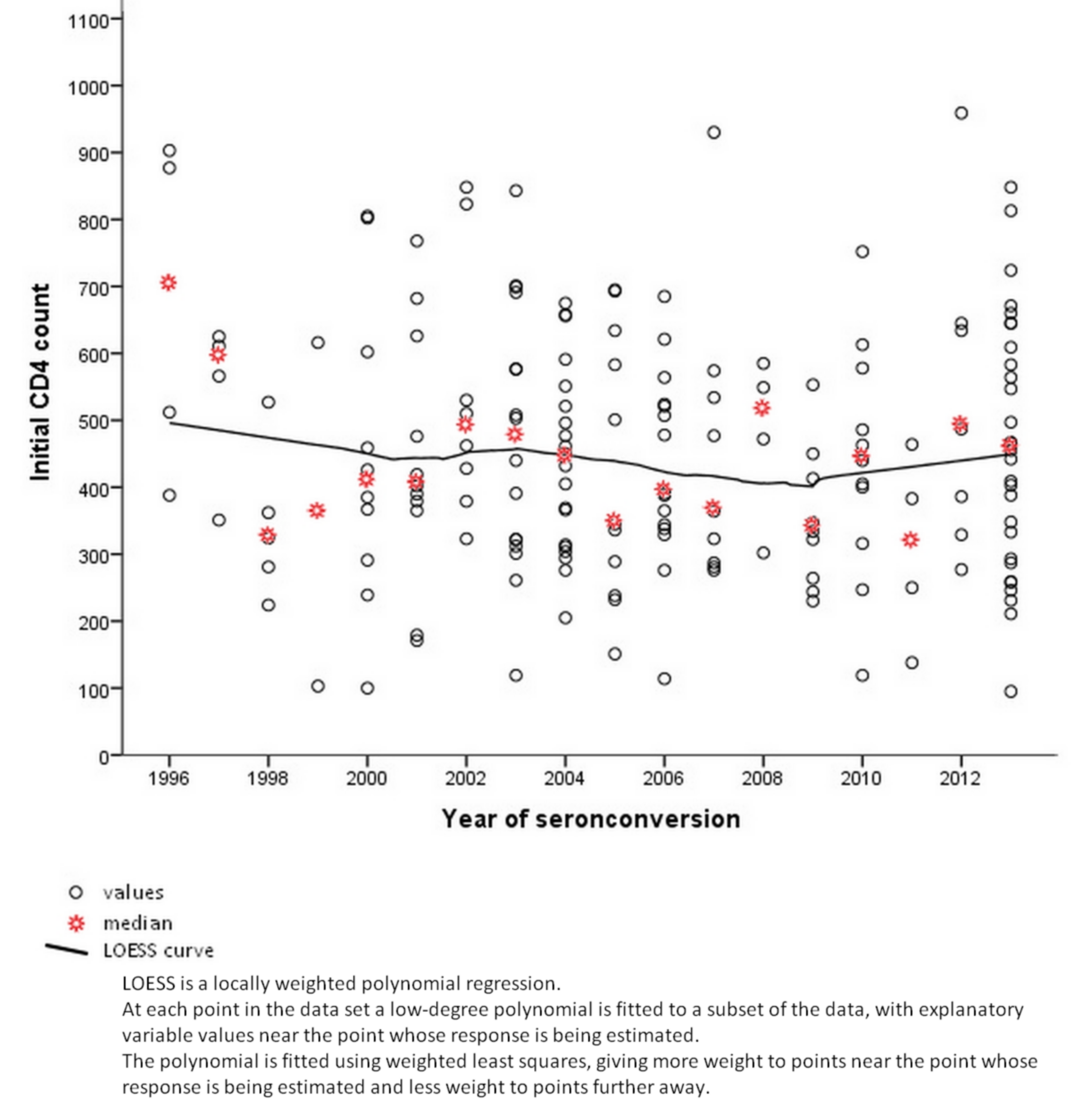

Supplement: S2 Fig — (TIFF) [file pone.0146978.s002.tiff]

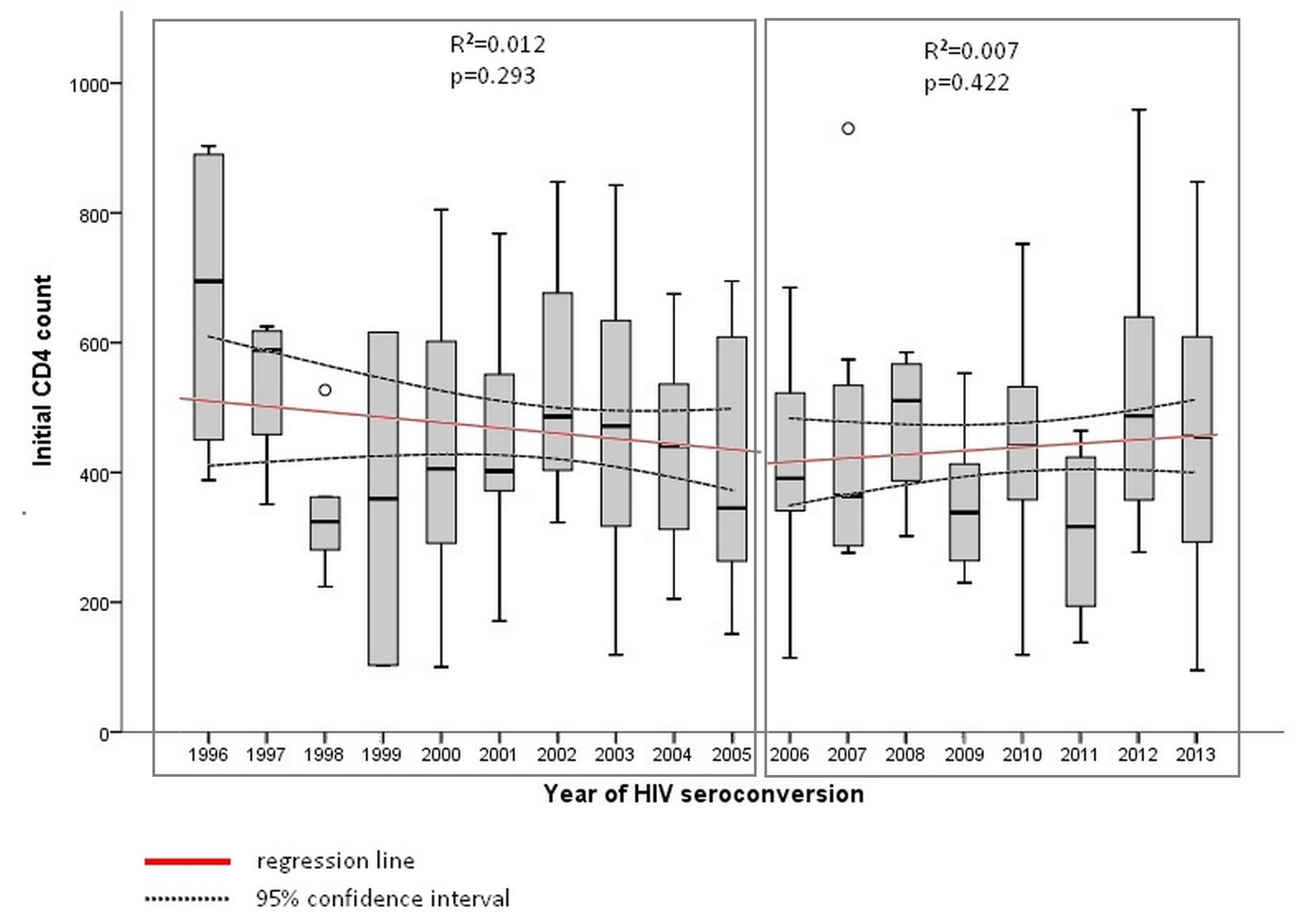

Supplement: S3 Fig — (TIFF) [file pone.0146978.s003.tiff]

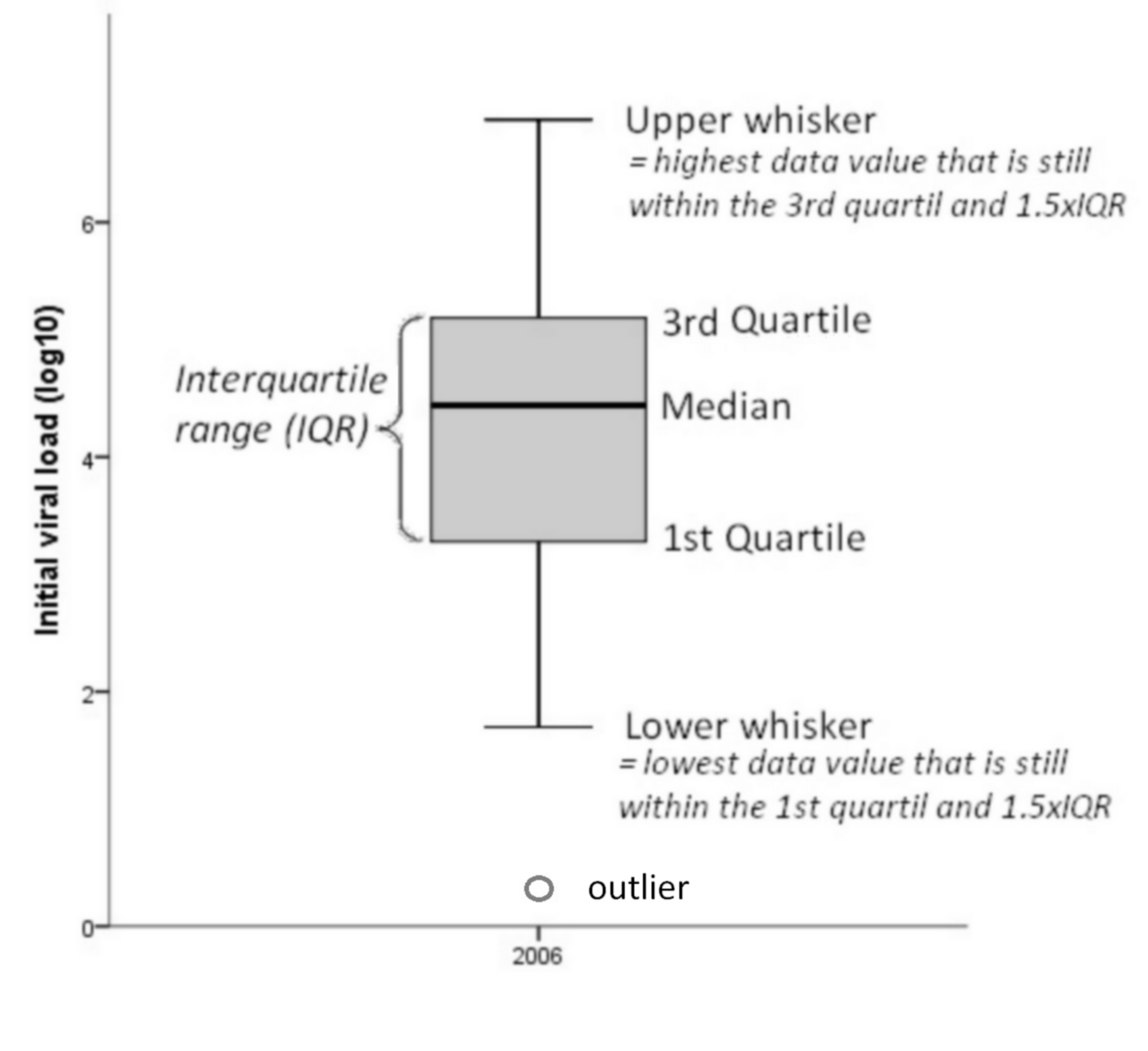

Supplement: S4 Fig — (TIFF) [file pone.0146978.s004.tiff]
